# Azimuth Full-Aperture Processing of Spaceborne Squint SAR Data with Block Varying PRF

**DOI:** 10.3390/s22239328

**Published:** 2022-11-30

**Authors:** Zhuo Zhang, Wei Xu, Pingping Huang, Weixian Tan, Zhiqi Gao, Yaolong Qi

**Affiliations:** 1 College of Information Engineering, Inner Mongolia University of Technology, Hohhot 010051, China; 2 Inner Mongolia Key Laboratory of Radar Technology and Application, Hohhot 010051, China

**Keywords:** synthetic aperture radar (SAR), squint sliding spotlight, pulse repetition frequency (BV-PRF), azimuth resampling, azimuth data reconstruction

## Abstract

The block varying pulse repetition frequency (BV-PRF) scheme applied to spaceborne squint sliding-spotlight synthetic aperture radar (SAR) can resolve large-range cell migration (RCM) and reduce azimuth signal non-uniformity. However, in the BV-PRF scheme, different raw data blocks have different PRFs, and the raw data in each block are insufficiently sampled. To resolve the two problems, a novel azimuth full-aperture pre-processing method is proposed to handle the SAR raw data formed by the BV-PRF scheme. The key point of the approach is the resampling of block data with different PRFs and the continuous splicing of azimuth data. The method mainly consists of four parts: de-skewing, resampling, azimuth continuous combination, and Doppler history recovery. After de-skewing, the raw data with different PRFs can be resampled individually to obtain a uniform azimuth sampling interval, and an appropriate azimuth time shift is introduced to ensure the continuous combination of the azimuth signal. Consequently, the resulting raw data are sufficiently and uniformly sampled in azimuth, which could be well handled by classical SAR-focusing algorithms. Simulation results on point targets validate the proposed azimuth pre-processing approach. Furthermore, compared with methods to process SAR data with continuous PRF, the proposed method is more effective.

## 1. Introduction

Spaceborne synthetic aperture radar (SAR) is an indispensable imaging technology for acquiring two-dimensional (2-D) high-resolution images of the Earth’s surface [1]. High-resolution spaceborne SAR has been widely applied to ship detection [2,3,4,5] in both civilian and military marine monitoring tasks [6,7,8] such as illegal stowaway, maritime management and coastal defense reconnaissance. The geometric resolution is one of the most important aspects of spaceborne SAR. The sliding-spotlight mode [9], which is achieved by steering the azimuth beam from fore toward aft to make the illuminated area move with a speed less than the radar platform, can apparently extend the synthetic aperture time to improve the azimuth resolution. However, in addition to the desired higher azimuth resolution, some areas are required to be clarified for future spaceborne SAR missions [10]. The squint sliding-spotlight mode [11,12] can obtain multiple images of a desired area with a fine azimuth resolution and different observation angles [13,14], and it will be widely adopted then future spaceborne SAR missions.

Usually, the sliding-spotlight SAR system working with a large squint angle will induce large-range cell migration (RCM) [15]. Many researchers have carried out a series of studies on the mentioned problem, but some issues still exist. Firstly, echo data with fixed pulse-repetition frequency (PRF) cannot be fully obtained, and the effective range swath width would be obviously reduced [16,17,18,19]. To resolve this problem, in [20], a continuous PRF variation scheme is proposed to achieve high-resolution wide-swath imaging. The continuously varying PRF (CV-PRF) technology applied to the squint SAR is proposed in [21] to resolve the effect of the large RCM on the reduced swath width. However, there are too many azimuth sampling intervals in the CV-PRF transmission scheme, which makes the subsequent azimuth signal uniform reconstruction difficult and require a lot of computing resources. Furthermore, the block varying PRF (BV-PRF) transmission scheme is proposed in [22] to avoid the above mentioned two problems simultaneously. The existing BV-PRF scheme research applied to squint SAR only involves the analysis of the basic principle; there is no complete theoretical research on its specific design and application. Specifically, in the squint sliding-spotlight mode, when the squint angle increases/decreases, the whole echo data of the imaged swath moves forward/backward in the echo-receiving window within a single pulse repetition interval (PRI). If the scattered echoes of the whole swath are received completely in the echo-receiving window within a single PRI, its corresponding PRF value remains unchanged, and the pulse transmission with the same sampling interval forms a burst block. If some of the scattered echoes move out of the front edge or trailing edge of the available echo-receiving window, its corresponding PRF value will be changed to obtain all the scattered echoes of the whole imaged swath. Since the instantaneous echo-receiving duration of the whole imaged swath in the spaceborne squint sliding-spotlight mode is relatively small and the RCM caused by the steering squint angle during the whole acquisition interval is much larger [23], the CV-PRF transmission scheme is no longer appropriate. Therefore, compared with the fixed PRF scheme, the BV-PRF transmission scheme makes the position of the blind area change in blocks along the azimuth direction; thus, it can solve the obviously reduced swath width in the fixed PRF scheme. Furthermore, compared with the CV-PRF scheme [24,25], the BV-PRF transmission scheme makes full use of the advantages of the relatively small instantaneous echo duration and the relatively large pulse repletion interval in spaceborne squint sliding-spotlight SAR, which can also greatly reduce the non-uniformity of azimuth signal in the CV-PRF scheme [26].

In this paper, the concrete design of the BV-PRF scheme applied to the spaceborne squint sliding-spotlight mode is proposed. The design of the BV-PRF scheme is mainly divided into four steps: (1) initial PRF setting, (2) the calculation of the instantaneous echo-receiving window position, (3) the determination of the sampling frequency range during azimuth beam steering and (4) judgment criterion and sampling frequency increment design. However, the azimuth processing for echo data formed by BV-PRF scheme brings two problems. Firstly, the BV-PRF scheme will cause different azimuth data blocks to arise with different PRFs. Secondly, the Doppler spectrum aliasing caused by the squint angle and azimuth beam steering in each data block will be introduced in the 2-D frequency domain [27,28,29]. To resolve the above two problems, a novel azimuth pre-processing method is proposed, and the key point of the method is the resampling of block data with different PRFs and the continuous splicing of azimuth data. However, the azimuth sampling frequency of each data block is insufficient due to the squint angle and azimuth beam steering. Firstly, de-skewing and de-ramping in the range frequency domain are performed to eliminate 2-D spectrum skewing caused by the squint angle and the extended bandwidth caused by azimuth beam steering, respectively. Afterwards, the total Doppler bandwidth of the raw data in each block is completely limited within the designed azimuth sampling frequency. Consequently, azimuth data in each block are resampled to obtain the same uniform azimuth sampling interval and facilitate the following azimuth data combination. Furthermore, an appropriate azimuth time shift should be introduced to ensure the continuity of the whole azimuth signal. Finally, re-skewing is introduced to recover the skewed 2-D spectrum. Compared with azimuth reconstruction methods of NUDFT [30], BLU [31], sinc interpolation [32] and multi-channel reconstruction method [33] with the CV-PRF scheme, the proposed azimuth pre-processing method for the squint sliding-spotlight SAR raw data with BV-PRF scheme is more efficient, since the proposed method uses only complex multiplication and fast Fourier transform (FFT) operations, without any matrix inversion and interpolation operations. Therefore, the proposed azimuth reconstruction method has the advantages of low computation, flexible processing and avoiding obvious system performances reduction.

This article is organized as follows. In Section 2, three pulse transmission sequences including fixed PRF, CV-PRF and BV-PRF in squint sliding-spotlight SAR are compared and discussed. The BV-PRF design is introduced in detail, and echo signal properties in the squint sliding-spotlight SAR are analyzed in Section 3. The proposed azimuth full-aperture pre-processing method for azimuth sufficient and uniform sampling is presented in Section 4. In Section 5, a simulation experiment on point targets is carried out to validate the proposed pre-processing method. Finally, this paper is discussed and concluded in Section 6 and Section 7.

## 2. Range Cell Migration Analysis for Squint SAR

**Figure 1:**
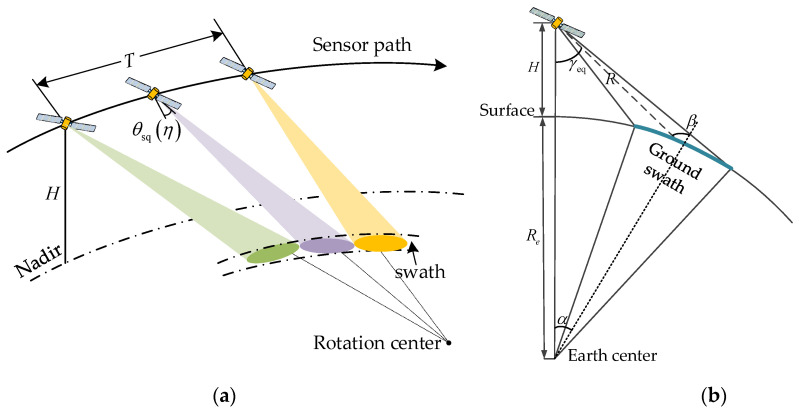
Geometric model of spaceborne squint sliding-spotlight SAR. (**a**) Squint-looking geometry; (**b**) side-looking geometry.

The geometric model of squint sliding-spotlight SAR is shown in Figure 1. Assuming that the azimuth beam scanning direction from aft to fore is in position, the instantaneous squint angle $\theta_{\mathrm{sq}}\left(\eta \right)$ changes from large to small during the whole acquisition interval T as shown in Figure 1a. In Figure 1b, *H* is the track height, $R_{e}$ is the earth radius, η is azimuth time, θ is the looking angle, γ is the incident angle, α is the geocentric angle, and R is the distance from the radar to the target.

As shown in Figure 1, the range history from radar to target can be calculated as:

(1) $$R\left(\gamma ,\eta ,\Delta \theta_{a}\right)=\sqrt{{\left(R_{e}+H\right)}^{2}+R_{e}^{2}-2R_{e}\left(H+R_{e}\right)\cos\left(\alpha \left(\gamma ,\eta ,\Delta \theta_{a}\right)\right)}$$

with

(2) $$\alpha \left(\gamma ,\eta ,\Delta \theta_{a}\right)=\gamma_{\mathrm{eq}}\left(\gamma ,\eta ,\Delta \theta_{a}\right)-\beta \left(\gamma ,\eta ,\Delta \theta_{a}\right)$$

(3) $$\beta \left(\gamma ,\eta ,\Delta \theta_{a}\right)=\arcsin\left(\sin\left(\gamma_{\mathrm{eq}}\left(\gamma ,\eta ,\Delta \theta_{a}\right)\right)\frac{R_{e}+H}{H}\right)$$

(4) $$\gamma_{\mathrm{eq}}\left(\gamma ,\eta ,\Delta \theta_{a}\right)=\arccos\left(\cos\gamma \cos\left(\theta_{\mathrm{sq}}+\omega_{r}\eta +\Delta \theta_{a}\right)\right)$$

where $\gamma \in \left[\gamma_{\mathrm{near}},\gamma_{\mathrm{far}}\right]$ is the looking angle, $\gamma_{\mathrm{near}}$ and $\gamma_{\mathrm{far}}$ are the near and far looking angles, respectively, $\theta_{\mathrm{sq}}$ is the squint angle in the middle of the acquisition interval, $\omega_{r}$ is the azimuth beam rotation rate, $\Delta \theta_{a}\in \left[-\theta_{a}/2,\theta_{a}/2\right]$ indicates the relative position in the illuminated azimuth beam, and $\theta_{a}$ is the exploited azimuth beam interval.

The echo duration of the whole desired range swath at any azimuth time η can be expressed as follows:

(5) $$\tau_{\mathrm{echo}}\left(\eta \right)=\tau_{r}+\frac{2}{c}\cdot \left[R\left(\gamma_{\mathrm{far}},\theta_{a}/2;\eta \right)-R\left(\gamma_{\mathrm{near}},-\theta_{a}/2;\eta \right)\right]$$

where $\tau_{r}$ is the transmitted pulse duration. The echo duration during the whole azimuth acquisition interval can be computed as ·

(6) $$\begin{array}{ll} \tau_{\mathrm{whole}} & =\tau_{r}+\frac{2}{c}\cdot \left[\max\left(R\left(\gamma_{\mathrm{far}},\theta_{a}/2;\eta \right)\right)-\min\left(R\left(\gamma_{\mathrm{near}},-\theta_{a}/2;\eta \right)\right)\right]\\ & =\tau_{r}+\frac{2}{c}\cdot \left[R\left(\gamma_{\mathrm{far}},\theta_{a}/2;-T/2\right)-R\left(\gamma_{\mathrm{near}},-\theta_{a}/2;T/2\right)\right] \end{array}$$

**Figure 2:**
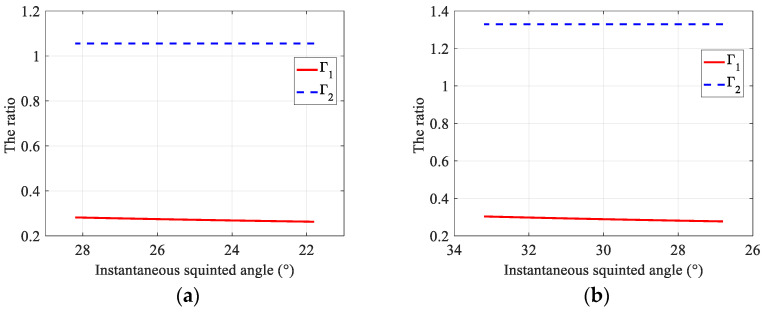
Comparison of $\Gamma_{1}$ and $\Gamma_{2}$ with different squint angles. (**a**) The squint angle in the middle acquisition interval is 25°; (**b**) the squint angle in the middle acquisition interval is 30°.

To demonstrate the large RCM caused by azimuth beam steering during the whole acquisition interval, two proportion factors, $\Gamma_{1}=\tau_{\mathrm{echo}}\cdot \mathrm{PRF}$ and $\Gamma_{2}=\tau_{\mathrm{whole}}\cdot \mathrm{PRF}$, are shown in Figure 2, where swath width is 20 km and $\gamma_{\mathrm{near}}$ is 32°. The factor $\Gamma_{1}$ demonstrates the ratio between the instantaneous echo duration and the pulse repletion interval (PRI), while $\Gamma_{2}$ indicates the ratio between the whole echo duration and the PRI. As shown in Figure 2, the instantaneous echo duration is much smaller than the PRI, and the whole echo duration of the swath would exceed the PRI, especially for the large squint angle.

**Figure 3:**
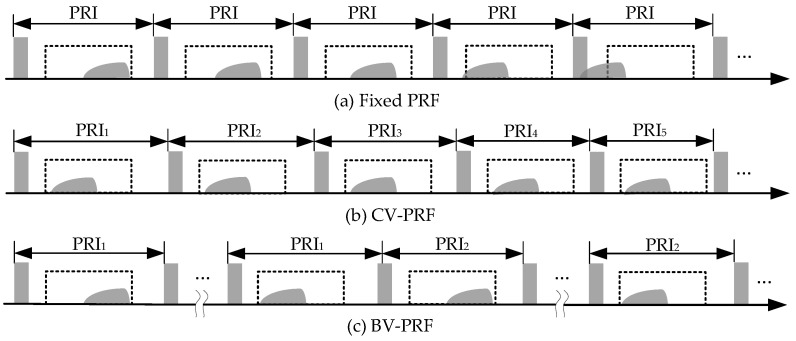
Diagrams of pulse transmission sequences. (**a**) Fixed PRF. (**b**) CV-PRF. (**c**) BV-PRF.

Using the fixed PRF sequence to obtain the squint sliding-spotlight mode data, it is easy to slide out the receiving window, as shown in Figure 3a. The CV-PRF transmission sequence was proposed to resolve the large RCM caused by large squint angle in the squint sliding-spotlight mode, and its operated PRF varies continuously with the squint angle to keep the instantaneous echo in almost the same position, as shown in Figure 3b. However, because of the energy constraint in the spaceborne mission, the swath width in the sliding-spotlight mode is usually a little more than 10 km, and this CV-PRF transmission scheme is not necessary in the squint sliding-spotlight mode. In addition, CV-PRF has various sampling frequencies in azimuth, which requires complex computation for subsequent reconstruction to obtain uniform sampled azimuth signal. The BV-PRF transmission scheme, which is a compromise between the fixed PRF and CV-PRF schemes, as shown in Figure 3c, can extend the imaged swath and reduce the non-uniformity of azimuth signal in the CV-PRF scheme simultaneously. Assuming that the central squint angle in the sliding-spotlight mode is positive, the RCM value gradually decreases, and the echo data move forward along the receiving window while steering the azimuth beam from fore toward aft. When the front edge of echo data do not reach the trailing edge of the transmitted pulse, the same PRF value is adopted; otherwise, the operated PRF is changed in order to prevent the desired echo data moving out the receiving window. In terms of the non-uniformity, the BV-PRF scheme is weaker than the CV-PRF scheme, and the echo data of the whole imaged swath can be acquired with a limited number of PRFs. Consequently, for the BV-PRF transmission scheme, the azimuth processing flexibility of echo data would be greatly improved.

## 3. PRF Design and Signal Analysis

### 3.1. Design of the BV-PRF Scheme

**Figure 4:**
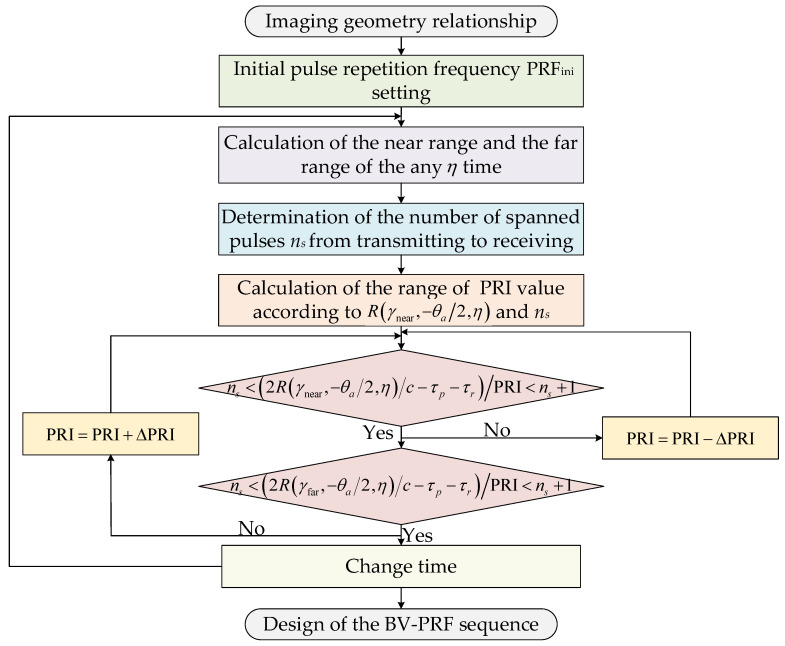
Flowchart of the design of the BV-PRF sequence.

The flowchart of the design of the BV-PRF sequence in the spaceborne squint sliding-spotlight mode is shown in Figure 4. As mentioned, the BV-PRF design adds a criteria equation for whether the scattered echo front edge exceeds the receiving window to control the variation of PRF with the squint angle. At first, according to the requirements of the range swath width and azimuth resolution, the initial pulse repetition frequency ${\mathrm{PRF}}_{\mathrm{ini}}$ of the system should be determined. The initial ${\mathrm{PRF}}_{\mathrm{ini}}$ determined by the Doppler bandwidth $B_{a}$ can be calculated as follows:

(7) $${\mathrm{PRF}}_{\mathrm{ini}}=\alpha_{s}\cdot \frac{1.772v_{s}\cos\theta_{\mathrm{sq}}}{L_{a}}$$

where $\alpha_{s}$ is the azimuth oversampling factor, $v_{s}$ is the platform speed, and $L_{a}$ is the azimuth antenna length.

Afterwards, the minimum value of $R\left(\gamma_{\mathrm{near}},-\theta_{a}/2;\eta \right)$ can be determined. Considering the case in which the echo data are received after the $n_{s}$ transmitted pulses are transmitted, the number $n_{s}$ can be expressed as follows:

(8) $$n_{s}=\left\lfloor \frac{2\min\left(R\left(\gamma_{\mathrm{near}},-\theta_{a}/2;\eta \right)\right)}{c}\cdot {\mathrm{PRF}}_{\mathrm{ini}}\right\rfloor$$

where ⌊·⌋ denote the floor operation, and c is the light speed.

Afterwards, the range of PRI will need to be determined according to the following:

(9) $$\begin{array}{l} {\mathrm{PRI}}_{\max}=\frac{2\cdot \max\left(R\left(\gamma_{\mathrm{near}},-\theta_{a}/2;\eta \right)\right)/c-\tau_{r}-\tau_{p}}{n_{s}}\\ {\mathrm{PRI}}_{\min}=\frac{2\cdot \min\left(R\left(\gamma_{\mathrm{near}},-\theta_{a}/2;\eta \right)\right)/c-\tau_{r}-\tau_{p}}{n_{s}} \end{array}$$

where $\tau_{p}$ is the guard interval.

As the squint angle gradually changes from large to small, the pulse repetition interval of the system changes from ${\mathrm{PRI}}_{\max}$ to ${\mathrm{PRI}}_{\min}$, and the range of pulse repetition frequency is $\left[{\mathrm{PRF}}_{\min},{\mathrm{PRF}}_{\max}\right]$. Assuming that the initial scanning angle is 28.2° and the terminal scanning angle is 21.8°, the position of echo data in the receiving window gradually moves forward during azimuth beam scanning. The pulse interval PRI remains unchanged if the pulse signal transmitted by the radar meets the following condition:

(10) $$n_{s}<\frac{2\cdot R\left(\gamma_{\mathrm{near}},-\theta_{a}/2;\eta \right)/c-\tau_{r}-\tau_{p}}{\mathrm{PRI}}<\left(n_{s}+1\right)$$

When the front edge of reflected echo arrives at the front edge of the receiving window, the judgment condition of (10) will be not applicable. Therefore, the value of PRI will need to be decreased by ΔPRI to meet the judgment condition corresponding to the pulse repetition interval PRI−ΔPRI; the judgment condition can be rewritten as follows:

(11) $$n_{s}<\frac{2\cdot R\left(\gamma_{\mathrm{near}},-\theta_{a}/2;\eta \right)/c-\tau_{r}-\tau_{p}}{\mathrm{PRI}-\Delta \mathrm{PRI}}<\left(n_{s}+1\right)$$

It should be noted that ΔPRI cannot exceed ${\mathrm{PRI}}_{\max}-{\mathrm{PRI}}_{\min}$.

**Figure 5:**
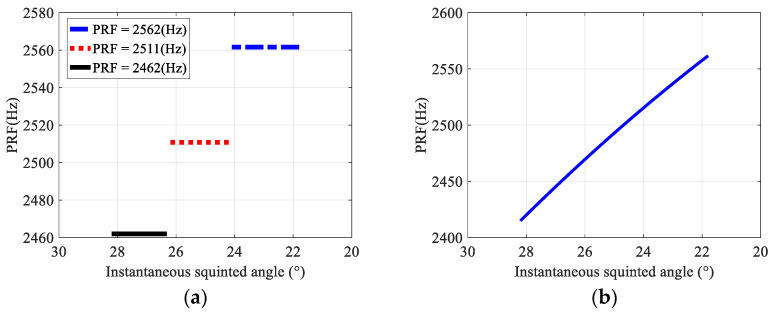
Design results of BV-PRF and CV-PRF. (**a**) BV-PRF; (**b**) CV-PRF.

PRF design results of BV-PRF and CV-PRF are given in Figure 5. As shown in Figure 5a, the number of PRFs in the BV-PRF transmitting scheme is 3, and the scanning angle ranges corresponding to 2462 Hz, 2511 Hz and 2562 Hz are 26.3°~28.2°, 24.2°~26.2° and 21.8°~24.1°, respectively. While the operated PRF of CV-PRF scheme changes continuously from the initial scanning angle 28.2°to the terminal scanning angle 21.8°, as shown in Figure 5b, and when the squint angle is about 26.3°, the PRF is 2462 Hz; when the squint angle is about 24.2°, the PRF is 2511 Hz.

**Figure 6:**
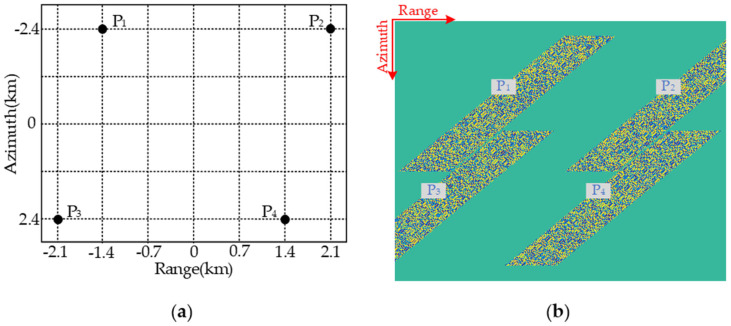

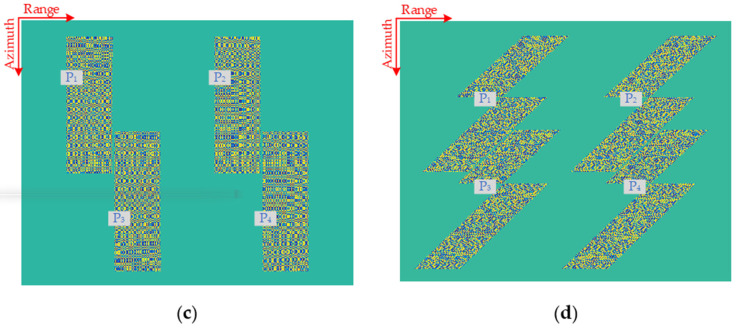
Echo simulation with different pulse transmission sequences. (**a**) Scene distribution of targets; (**b**) the fixed PRF; (**c**) the CV-PRF; (**d**) the BV-PRF.

In order to validate the raw-data-obtaining capacities of both BV-PRF and CV-PRF transmitting schemes, SAR raw data simulation experiments were carried out, and the designed scene is shown in Figure 6a. SAR raw data simulation results with fixed PRF, CV-PRF and BV-PRF are shown in Figure 6b–d, respectively. As the RCM is increased rapidly with the squint angle, the SAR raw data with the fixed PRF of the whole imaged scene cannot be fully obtained for targets located in the edge of the swath, as shown in Figure 6b. The distortion of the raw data with CV-PRF is removed, and the resulting raw data are shown in Figure 6c. However, the continuously varying PRF makes the subsequent azimuth signal uniform reconstruction difficult. The raw data of the whole scene with BV-PRF scheme can be obtained by changing PRF three times, as shown in Figure 6d. The BV-PRF takes advantage of instant short echo duration time to reduce the number of PRF changes, which makes following azimuth data reconstruction easy.

### 3.2. Properties of Echo Signal with BV-PRF

**Figure 7:**
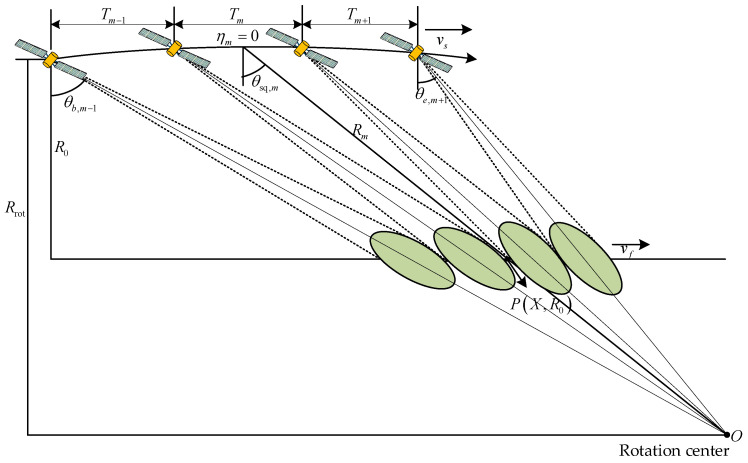
The imaging geometry of spaceborne squint sliding-spotlight SAR with BV-PRF.

The imaging geometry of spaceborne squint sliding-spotlight SAR data with BV-PRF is illustrated in Figure 7. During the whole raw data acquisition interval, the azimuth beam scanning from front to back makes the beam footprint move with a speed less than the radar platform. $\theta_{\mathrm{sq},m}$ is the central squint angle of the *m*-th data block, P is a point target located in the position $\left(X,\;R_{0}\right)$ in the imaged swath, $R_{0}$ and $R_{\mathrm{rot}}$ are the slant ranges from the sensor path to the imaged target and the virtual rotation center, respectively, and $T_{m}$ is the whole acquisition interval of data block corresponding to ${\mathrm{PRF}}_{m}$. The azimuth beam scanning at a constant rotation rate $\omega_{r}$ leads to a steering factor *A*. It is defined as follows:

(12) $$A(R_{0})\approx \frac{R_{\mathrm{rot}}-R_{0}}{R_{\mathrm{rot}}}=\frac{v_{f}(R_{0})}{v_{g}}$$

where $v_{g}$ is the ground velocity, and $v_{f}$ is the moving speed of the azimuth antenna beam center in the imaged scene.

The third-order Taylor expansion of instantaneous slant range $R_{m}(\eta_{b,m})$ can be written as follows:

(13) $$R_{m}\left(\eta_{b,m}\right)\approx R_{c}-\frac{\lambda }{2}\cdot f_{\mathrm{dc},m}\left(\eta_{b,m}-\eta_{x}\right)-\frac{\lambda }{4}\cdot K_{a,m}{\left(\eta_{b,m}-\eta_{x}\right)}^{2}+\frac{\lambda }{4}p_{\mathrm{cubic},m}{\left(\eta_{b,m}-\eta_{x}\right)}^{3}\cdots$$

with

(14) $$K_{a,m}=-\frac{2v_{s}^{2}\cos^{3}\theta_{\mathrm{sq},m}}{\lambda R_{0}}$$

where $R_{c}=R_{0}/\cos\left(\theta_{\mathrm{sq},m}\right)$ is the slant range from the satellite platform to the center of the imaged scene, $\eta_{b,m}={\left(-N_{a,m}/2,\ldots ,N_{a,m}/2-1\right)/\mathrm{PRF}}_{m}+t_{m}$, where $N_{a,m}$ is the number of azimuth samples of the *m*-th azimuth block and $t_{m}$ is the time shift of the azimuth time of the *m*-th block data relative to the entire azimuth signal, $\eta_{x}=X/v_{g}$ shows the azimuth position of the target, and $p_{\mathrm{cubic},m}$ is the coefficient of the cubic term for the slant range expansion.

The azimuth signal of point target $P\left(X,\;R_{0}\right)$ of the squint sliding-spotlight mode corresponding to the *m*-th block data is expressed as [34]:

(15) $$\begin{array}{ll} s_{m}\left(\eta_{b,m};X,R_{0}\right) & =A_{0}\cdot \mathrm{rect}\left[\frac{\eta_{b,m}-\eta_{x}/A}{T_{f}/A}\right]\cdot \exp\left(-j\frac{4\pi }{\lambda }\frac{R_{0}}{\cos\theta_{\mathrm{sq},m}}\right)\\ & \cdot \exp\left[j\frac{4\pi v_{s}\sin\theta_{\mathrm{sq},m}}{\lambda }\left(\eta_{b,m}-\eta_{x}\right)-j\pi \frac{2v_{s}^{2}\cos^{3}\theta_{\mathrm{sq},m}}{\lambda R_{0}}{\left(\eta_{b,m}-\eta_{x}\right)}^{2}\right] \end{array}$$

The cubic-order term in (13) is neglected for simplicity and without losing the rationale of the discussion, and the cubic-order term is still compensated in the following 1-D azimuth signal analysis. Using the principle of stationary phase (POSP), the azimuth signal spectrum can be expressed as:

(16) $$\begin{array}{ll} S_{m}\left(f_{\eta ,m};X,R_{0}\right)= & A_{1}\cdot \mathrm{rect}\left[\frac{f_{\eta ,m}-f_{\mathrm{dc},m}+\left(A-1\right)\cdot K_{a}\eta_{x}/A}{B_{f}/A}\right]\cdot \exp\left(-j\frac{4\pi }{\lambda }\frac{R_{0}}{\cos\theta_{\mathrm{sq},m}}\right)\\ & \cdot \exp\left(-j\pi \frac{f_{\eta ,m}^{2}}{K_{a}}+j2\pi f_{\eta ,m}\frac{f_{\mathrm{dc},m}}{K_{a}}-j2\pi f_{\eta ,m}\eta_{x}+j2\pi f_{\eta ,m}t_{m}\right) \end{array}$$

where $A_{1}$ is a complex constant, $f_{\eta ,m}$ is the Doppler frequency of the *m*-th data block, and $B_{f}$ is the azimuth beam bandwidth exploited for azimuth focusing. In the squint sliding-spotlight SAR, the total Doppler bandwidth will be increased owing to the azimuth beam scanning, and the instantaneous azimuth beam Doppler center varying rate $k_{\mathrm{rot},m}$ of the *m*-th data block is given as follows:

(17) $$k_{\mathrm{rot},m}\left(f_{\tau }\right)\approx \frac{2v_{s}\left(f_{c}+f_{\tau }\right)\omega_{r}\cos\left(\theta_{\mathrm{sq},m}+\omega_{r}\eta_{b,m}\right)}{c}$$

**Figure 8:**
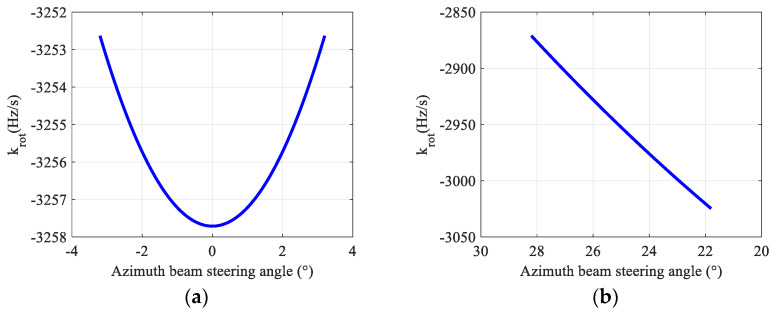
Variation curves of the instantaneous Doppler centroid varying rate $k_{\mathrm{rot}}$ under the side-looking and the squint. (**a**) $k_{\mathrm{rot}}$ within ±3.2 under the side-looking; (**b**) $k_{\mathrm{rot}}$ within ±3.2 under the squint angle 25°.

According to (17), variation curves of the instantaneous Doppler center varying rate within an appropriate azimuth beam scanning range under the side-looking and the squint are shown in Figure 8, respectively.

It can be seen in Figure 8 that when the squint angle is 0°, the change in instantaneous Doppler center varying rate $k_{\mathrm{rot}}$ can be ignored; when the squint angle is 25°, the change in instantaneous Doppler center varying rate $k_{\mathrm{rot}}$ reaches 160 Hz/s. Therefore, as the squint angle changes, the change in $k_{\mathrm{rot}}$ needs to be considered and changes nonlinearly within an appropriate azimuth beam scanning range.

The Doppler frequency $f_{\eta ,m}$ in the *m*-th data block can be computed as:

(18) $$f_{\eta ,m}\left(f_{\tau },\eta_{b,m},\Delta \theta_{a}\right)=\frac{2v_{s}\cdot \left(f_{c}+f_{\tau }\right)\cdot \sin\left(\theta_{\mathrm{sq},m}-\omega_{r}\eta_{b,m}-\Delta \theta_{a}\right)}{c}$$

where $f_{c}$ is the carrier frequency, and $f_{\tau }\in [-B_{r}/2,B_{r}/2]$ is the range frequency.

**Figure 9:**
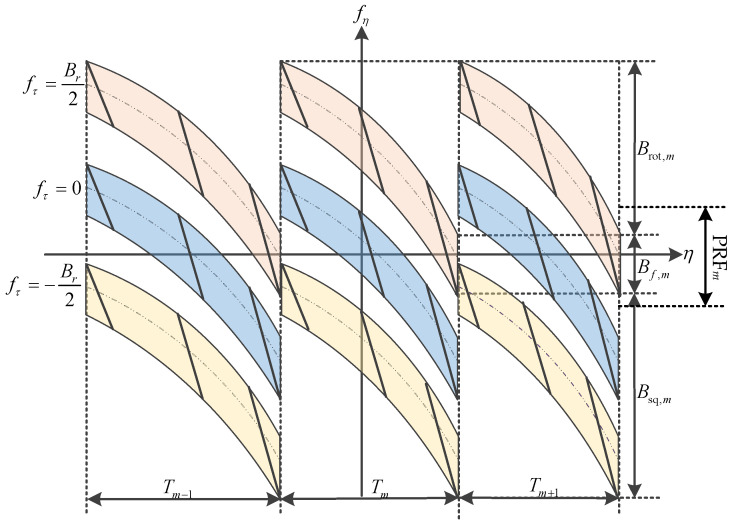
Azimuth time–frequency diagram of squint sliding-spotlight SAR data with BV-PRF.

According to (17) and (18), the azimuth time–frequency diagram of spaceborne squint sliding-spotlight SAR data with BV-PRF is shown in Figure 9.

**Figure 10:**
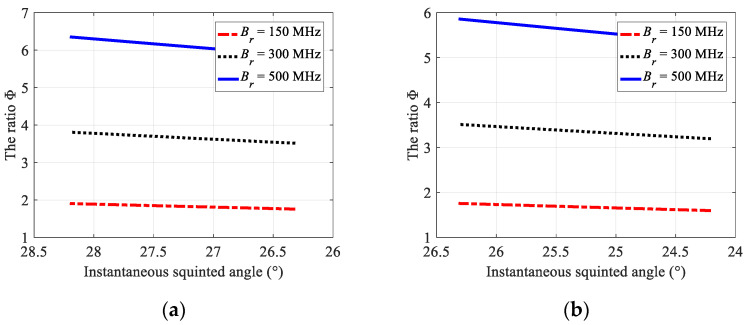
Ratios of the squint additional bandwidth to the azimuth beam bandwidth in adjacent data blocks. (**a**) The ratio Φ in the prior data block; (**b**) the ratio Φ in the latter data block.

The total Doppler bandwidth of the squint sliding-spotlight SAR data with BV-PRF can be computed as

(19) $$\begin{array}{ll} B_{\mathrm{tot},m} & =\frac{2v_{s}\cos\theta_{\mathrm{sq},m}}{\lambda }\theta_{a}+\int_{-T_{m}/2}^{T_{m}/2}\left|k_{\mathrm{rot},m}\right|d\eta_{b,m}+\frac{2v_{s}B_{r}}{c}\sin\theta_{\mathrm{sq},m}\\ & =B_{f,m}+B_{\mathrm{rot},m}+B_{\mathrm{sq},m} \end{array}$$

where $B_{r}$ is the transmitted pulse bandwidth. From (19), it can be seen that the total Doppler bandwidth of each block data is composed of three main parts: azimuth beam bandwidth $B_{f,m}$, extended Doppler bandwidth $B_{\mathrm{rot},m}$ caused by azimuth beam steering, and additional bandwidth $B_{\mathrm{sq},m}$ caused by the squint angle. In order to analyze the influence of bandwidth $B_{\mathrm{rot},m}$ and bandwidth $B_{\mathrm{sq},m}$ on the total Doppler bandwidth and azimuth spectrum aliasing in each raw data block, the ratio between the additional Doppler bandwidth to the azimuth beam bandwidth varying with the instantaneous squint angle under different pulse bandwidths is shown in Figure 10.

Generally, the azimuth over-sampling rate between PRF and azimuth beam bandwidth in spaceborne sliding-spotlight SAR is set to 1.3~1.5. When the transmitted pulse bandwidth is greater than 150 MHz, the sum of squinted additional bandwidth and azimuth beam bandwidth is greater than the azimuth sampling frequency of the system, as shown in Figure 10. For the prior data block with the central squint angle of 27.3°, when the pulse bandwidth is 150 MHz, the total Doppler bandwidth is 7176 Hz, which is greater than the operated PRF. For the latter data block with the central squint angle of 25.3°, when the pulse bandwidth is 150 MHz, the total Doppler bandwidth is 7225 Hz and is also greater than the PRF. Therefore, the Doppler spectrum aliasing of each block data caused by the squint angle and azimuth beam steering must be eliminated before azimuth combination.

## 4. Azimuth Pre-Processing

### 4.1. Azimuth Pre-Processing in the 1-D Domain

Because the cubic term function of azimuth time in the expansion of the range history will influence azimuth focusing, the azimuth signal of the *m*-th data block, which takes the cubic term function into account, can be rewritten as (neglecting the constants and azimuth amplitude weighting) [16]:

(20) $$s_{m}\left(\eta_{b,m}\right)=\exp\left(j\frac{4\pi v_{s}\sin\theta_{\mathrm{sq},m}}{\lambda }\eta_{b,m}-j\pi \frac{2v_{s}^{2}\cos^{3}\theta_{\mathrm{sq},m}}{\lambda R_{0}}\eta_{b,m}^{2}\right)\cdot \exp\left(-j\pi p_{\mathrm{cubic},m}\eta_{b,m}^{3}\right)$$

with

(21) $$p_{\mathrm{cubic},m}=\frac{2v_{s}{}^{3}\sin\left(\theta_{\mathrm{sq},m}\right)\cos^{4}\left(\theta_{\mathrm{sq},m}\right)}{\lambda R_{0}{}^{2}}$$

**Figure 11:**
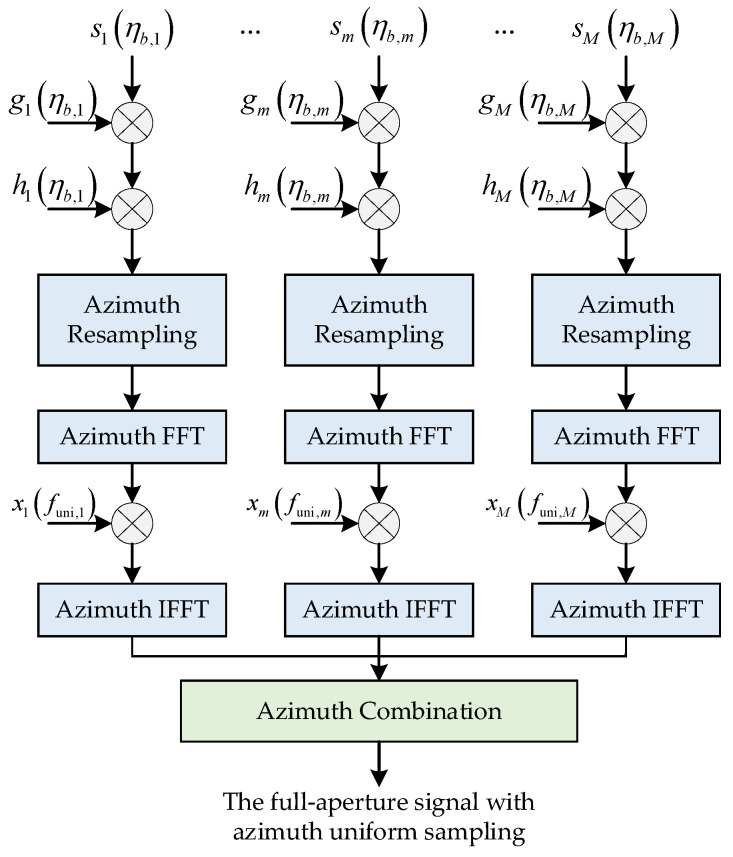
The block diagram of 1-D azimuth pre-processing of the proposed method.

The block diagram of 1-D azimuth signal processing for spaceborne squint sliding-spotlight SAR azimuth echo data generated by the designed BV-PRF scheme is shown in Figure 11, which mainly includes three parts: phase compensation, azimuth resampling and azimuth data combination. Firstly, the first- and third-term phase compensation should be performed in azimuth time domain. Afterwards, azimuth resampling operation is required to obtain the signal of azimuth uniform sampling. Furthermore, phase-shift compensation is executed in azimuth frequency domain to guarantee the continuous azimuth combination.

To eliminate the range walk term caused by the first term about azimuth time in (20), the phase compensation function $g_{m}\left(\eta_{b,m}\right)$ in the *m*-th data block is multiplied and expressed as follows

(22) $$g_{m}\left(\eta_{b,m}\right)=\exp\left(-j\frac{4\pi v_{s}\sin\theta_{\mathrm{sq},m}}{\lambda }\eta_{b,m}\right)$$

Afterwards, to carry out matched filtering successfully, the cubic term function about azimuth time in (20) must be compensated. The phase compensation function $h_{m}\left(\eta_{b,m}\right)$ for the *m*-th data block is as follows

(23) $$h_{m}\left(\eta_{b,m}\right)=\exp\left(j\frac{2\pi v_{s}{}^{3}\cos^{4}\left(\theta_{\mathrm{sq},m}\right)\sin\left(\theta_{\mathrm{sq},m}\right)}{\lambda R_{0}{}^{2}}\eta_{b,m}^{3}\right)$$

After linear and cubic term phase compensation, different data blocks with different PRFs need to be resampled to obtain uniform sampling rate. For continuous azimuth combination of different azimuth data blocks, the azimuth time shift should be introduced in the azimuth time domain after azimuth resampling. The azimuth time shift is operated in the Doppler domain and multiplied by the phase shift function as follows:

(24) $$x_{m}\left(f_{\mathrm{uni},m}\right)=\exp\left(j2\pi f_{\mathrm{uni},m}t_{\mathrm{uni},m}\right)$$

where $t_{\mathrm{uni},m}$ is the time shift of the time center of the *m*-th block data relative to the signal center of the whole bandwidth after the azimuth resampling, and $f_{\mathrm{uni},m}$ is the azimuth frequency after azimuth resampling.

**Figure 12:**
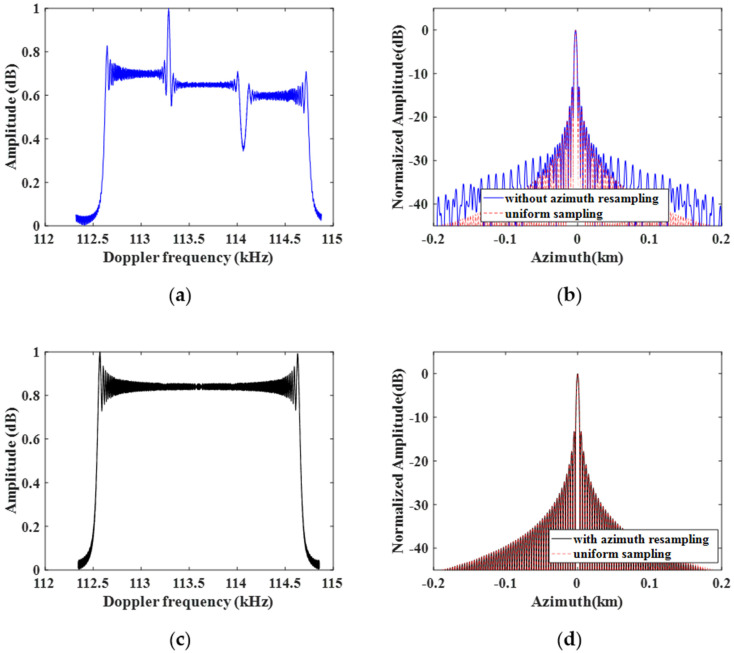
One-dimensional azimuth compression results of the proposed method. (**a**) Azimuth spectrum without azimuth resampling; (**b**) the azimuth compression result of (**a**); (**c**) Doppler spectrum after azimuth resampling; (**d**) the azimuth compression result of (**c**).

Figure 12 shows the results of 1-D azimuth compression without azimuth resampling and with azimuth resampling of the proposed method. Without removing the azimuth non-uniform sampling before the azimuth combination, the corresponding amplitude spectrum is discontinuous, as shown in Figure 12a, and the azimuth compression result shows pairs of false targets, as shown in Figure 12b. However, after the azimuth resampling for data block with different PRFs, the spectrum is well-reconstructed, and the false targets are suppressed, as shown in Figure 12c,d.

### 4.2. Azimuth Pre-Processing in the 2-D Domain

**Figure 13:**
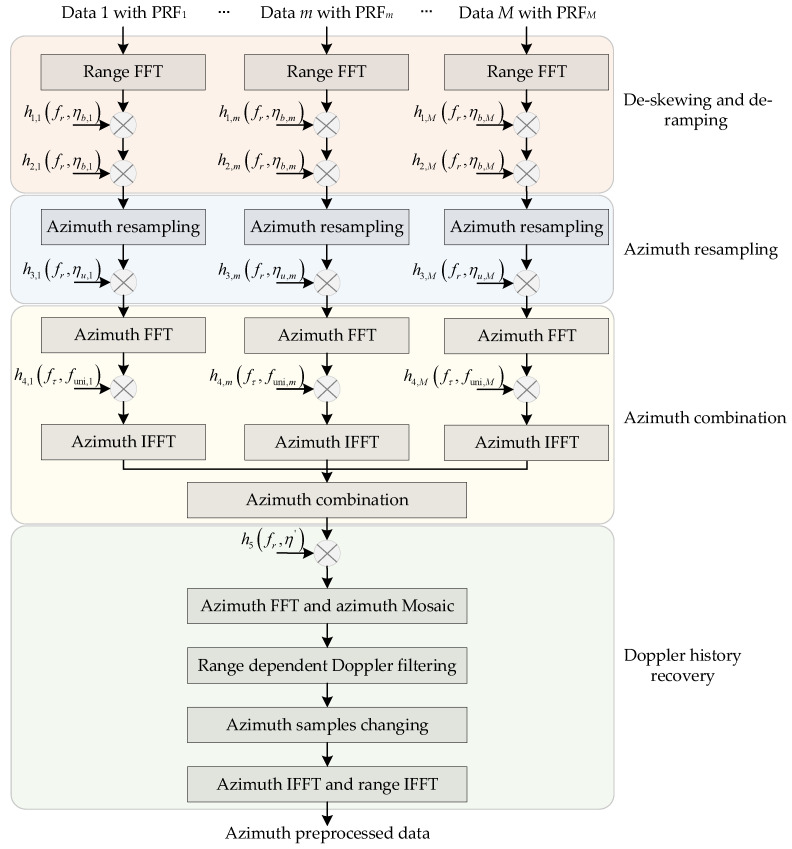
The block diagram of 2-D azimuth pre-processing of the proposed method.

The squinted additional bandwidth and beam rotation bandwidth of raw data with BV-PRF spaceborne squinted sliding-spotlight mode gradually increases with the squint angle, which makes the total Doppler bandwidth of each sub-block data span over several designed azimuth sampling frequencies. In addition, the BV-PRF scheme results in azimuth non-uniform sampling. However, the azimuth up-sampling technology, the traditional two-step pre-processing algorithm and full-aperture focusing method [14] will become inapplicable. To solve the above two problems, an azimuth pre-processing approach combining the BV-PRF and full-aperture processing is proposed in this section. The block diagram of the proposed pre-processing approach is shown in Figure 13. The proposed azimuth pre-processing method is mainly divided into four processing steps: range frequency dependent de-skewing and de-ramping, azimuth signal resampling, azimuth data combination and Doppler history recovery.

The 2-D echo signal of the *m*-th data block is expressed as follows:

(25) $$\begin{array}{ll} s_{\mathrm{block},m}\left(\tau ,\eta_{b,m};R_{m}\right) & =\mathrm{rect}\left[\frac{\tau -2R_{m}\left(\eta_{b,m}\right)/c}{T_{r}}\right]\mathrm{rect}\left[\frac{\eta_{b,m}-\eta_{x}/A}{T_{f}/A}\right]\\ & \cdot \exp\left(-j\frac{4\pi R_{m}\left(\eta_{b,m}\right)}{\lambda }\right)\exp\left(j\pi K_{r}{\left(\tau -2R_{m}\left(\eta_{b,m}\right)/c\right)}^{2}\right) \end{array}$$

where $K_{r}$ is the frequency modulation rate of the transmitted pulse.

Since the Doppler center of each data block changes with the range frequency, the spectrum in the 2-D frequency domain is skewed. This means that the de-skewing operation must be implemented in the range frequency domain. The following transfer function is multiplied after range Fourier transform in each sub-block to remove the Doppler bandwidth caused by the spaceborne squint angle:

(26) $$h_{1,m}\left(f_{\tau },\eta_{b,m}\right)=\exp\left\{-j2\pi \cdot \left(1+\frac{f_{\tau }}{f_{c}}\right)\cdot f_{\mathrm{dc},m}\cdot \eta_{b,m}\right\}$$

After the de-skewing processing, the distorted 2-D spectrum becomes flat, and the total Doppler bandwidth of squint sliding-spotlight SAR becomes the sum of the additional bandwidth introduced by the Doppler center varying and the azimuth beam bandwidth. When the duration time of an arbitrary data point in BV-PRF scheme is too long, the remaining Doppler bandwidth can be still greater than the sampling frequency of each block after the de-skewing. Therefore, the range frequency-dependent de-ramping operation must be performed. The de-ramping function can be shown as follows:

(27) $$h_{2,m}\left(f_{\tau },\eta_{b,m}\right)=\exp\left\{-j\pi k_{\mathrm{rot},m}\left(f_{\tau }\right)\cdot \eta_{b,m}^{2}\right\}$$

**Figure 14:**
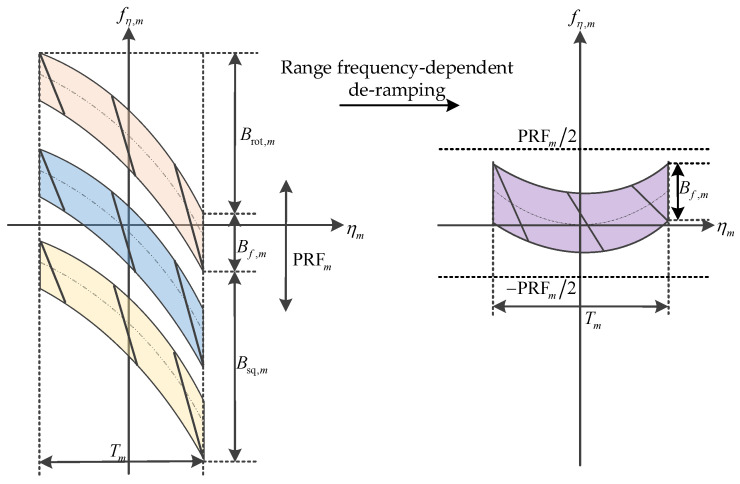
Azimuth time–frequency diagram after de-ramping processing.

After the range-frequency-dependent de-ramping processing, the total Doppler bandwidth of the spaceborne squint sliding-spotlight raw data with BV-PRF is limited within the sampling frequency PRF, as shown in Figure 14. Afterwards, the resampling operation, which is used to transform the azimuth non-uniform data block corresponding to different PRFs into uniform data, must be performed in order to smoothly combine each block data point in azimuth.

Because the range-frequency-dependent de-skewing and de-ramping processing of the signal will introduce additional phase terms, it is necessary to eliminate the redundant phase terms in the subsequent processing to restore the original Doppler history of the signal. Then, the re-ramping operation can be performed by multiplying the re-ramping function with the signal after the azimuth resampling of each sub-block data. The re-ramping function can be written as

(28) $$h_{3,m}\left(f_{\tau },\eta_{u,m}\right)=\exp\left\{j\pi k_{\mathrm{rot},m}\left(f_{\tau }\right)\cdot \eta_{u,m}^{2}\right\}$$

where $\eta_{u,m}=\eta_{\mathrm{uni},m}+t_{\mathrm{uni},m}$, $\eta_{\mathrm{uni},m}=\left(-n_{a,m}/2:n_{a,m}/2-1\right)/{\mathrm{PRF}}_{\mathrm{uni}}$, $n_{a,m}$ is the number of the signal sampling of the *m*-th block data after the azimuth resampling. ${\mathrm{PRF}}_{\mathrm{uni}}$ is the uniform sampling frequency after the azimuth resampling.

Since azimuth resampling introduces azimuth time shift in the azimuth time domain, it is necessary to phase shift in the azimuth frequency domain to continuously combine each block data in the azimuth. The phase shift function can be expressed as:

(29) $$h_{4,m}\left(f_{\mathrm{uni},m}\right)=\exp\left\{j2\pi f_{\mathrm{uni},m}t_{\mathrm{uni},m}\right\}$$

After the azimuth combination processing, the full-aperture data with azimuth uniform sampling will be obtained. Afterward, the Doppler histories should be recovered by multiplying the following re-skewing function:

(30) $$h_{5}\left(f_{\tau },\eta^{\prime }\right)=\exp\left\{j2\pi \cdot \frac{2v_{s}\sin\theta_{\mathrm{sq}}f_{\tau }}{c}\cdot \eta^{\prime }\right\}$$

where $\eta^{\prime }=\left(-N_{a}/2:N_{a}/2-1\right)/{\mathrm{PRF}}_{\mathrm{uni}}$, and $N_{a}$ is the number of the total signal sampling after the azimuth combination.

Different from the conventional sliding-spotlight mode, the total Doppler bandwidth of the complete raw data in the spaceborne squint sliding-spotlight mode after the re-skewing operation is still back-folded. Therefore, an azimuth data mosaic operation to resolve the problem of residual Doppler spectrum back-folding should be introduced.

At first, multiple replications of azimuth data are arranged together in the Doppler domain to resolve the aliased Doppler spectrum. The number of replications is

(31) $$Q_{f}=2\left\lceil \frac{B_{f}+B_{\mathrm{sq}}}{2\cdot {\mathrm{PRF}}_{\mathrm{uni}}}\right\rceil +1$$

where $B_{f}$ and $B_{\mathrm{sq}}$ are the azimuth beam bandwidth and the squint additional bandwidth after azimuth combination, respectively.

After the azimuth mosaic operation in the 2-D frequency domain [34], the following range-frequency-variant Doppler filter is applied to remove the redundant spectrum and to obtain the 2-D spectrum of the desired raw data.

(32) $$H(f_{\tau },f_{\eta })=\{\begin{array}{cc} 1, & \mathrm{with}\;\left|f_{\eta }-\frac{2\cdot f_{\tau }\cdot v_{s}}{c}\sin\theta_{\mathrm{sq}}\right|<\frac{{\mathrm{PRF}}_{\mathrm{uni}}}{2}\\ 0, & \mathrm{otherwise} \end{array}$$

with

(33) $$f_{\eta }\in \left[-\frac{Q_{f}\cdot {\mathrm{PRF}}_{\mathrm{uni}}}{2},\frac{Q_{f}\cdot {\mathrm{PRF}}_{\mathrm{uni}}}{2}\right]$$

After Doppler filtering, the Doppler spectrum of the original echo data without aliasing is obtained, but the number of refreshed azimuth samples is obviously increased. In order to improve the efficiency of the proposed algorithm, the redundant spectrum at both ends of azimuth frequency domain needs to be deleted, the new azimuth sampling frequency is updated as follows:

(34) $$\delta \cdot {\mathrm{PRF}}_{\mathrm{uni}}>\left[\left(B_{f}+B_{\mathrm{sq}}\right)/B_{f}\right]\cdot {\mathrm{PRF}}_{\mathrm{uni}}$$

Finally, the raw 2-D spectrum with sufficient sampling frequency is obtained.

## 5. Simulation Experiments

**Figure 15:**
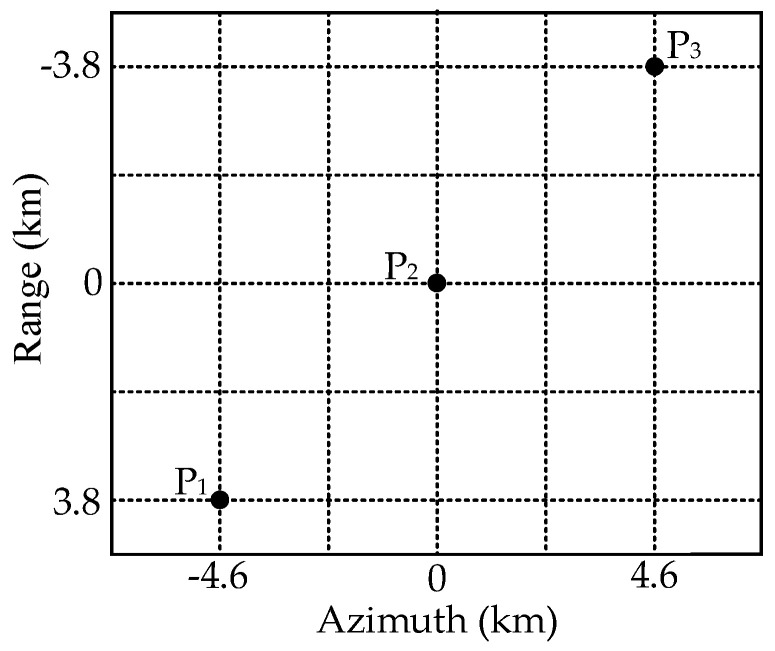
The designed imaging scene with three point targets.

In this section, a designed simulation experiment on three point targets is carried out to validate the proposed pre-processing method, and simulation parameters are shown in Table 1. The designed scene is shown in Figure 15, and the squint observation angle in the azimuth middle time is 25°.

**Figure 16:**
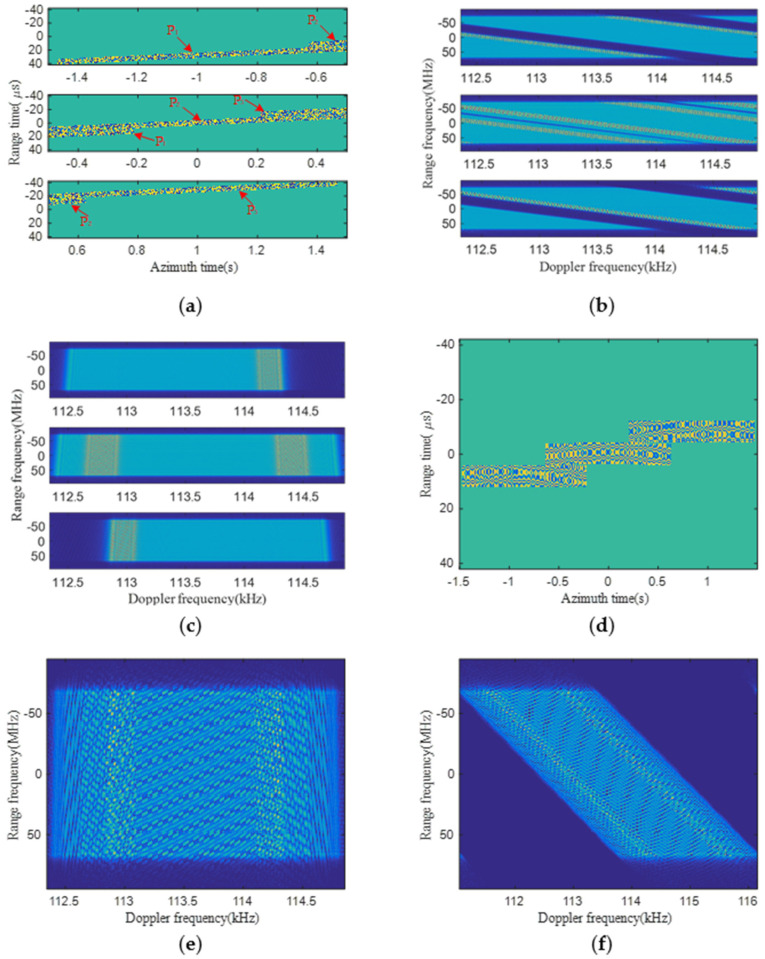
Simulation results of the proposed method. (**a**) The real part of echo data in three blocks; (**b**) 2-D spectra of (**a**); (**c**) 2-D spectra in three blocks before azimuth combination; (**d**) echo data of the whole imaged scene after azimuth combination; (**e**) 2-D spectrum of (**d**) before re-skewing; (**f**) the recovered 2-D spectrum.

The real parts of echo data of three point targets with different PRFs are shown in Figure 16a. Their corresponding 2-D spectra are shown in Figure 16b, and the 2-D spectrum of raw data in each block is aliased in the Doppler domain. Afterwards, the non-aliased 2-D spectrum of raw data in each block is obtained after the de-skewing, range-frequency-dependent de-ramping and azimuth resampling, as shown in Figure 16c. In Figure 16a–c, the first block contains the echo data and spectrum of P_1_ and P_2_; the middle block contains the echo data and spectrum of P_1_, P_2_ and P_3_; and the third block contains the echo and spectrum of P_2_ and P_3_. Consequently, the reconstructed signal of the whole scene in the 2-D time domain has uniform sampling frequency after azimuth combination, as shown in Figure 16d, and its corresponding spectrum is shown in Figure 16e. Finally, the original 2-D spectrum with sufficient sampling frequency is well-recovered by azimuth re-skewing and range-frequency-dependent filtering, as shown in Figure 16f.

**Figure 17:**
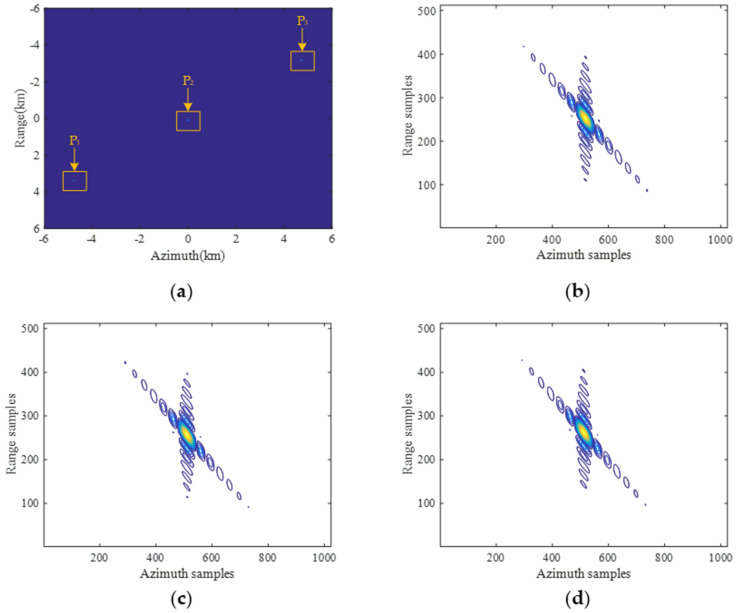
Imaging results on three point-targets handled by the proposed method. (**a**) Imaging results with three points; (**b**) contour plot of target P_1_; (**c**) contour plot of target P_2_; (**d**) contour plot of target P_3_.

The imaging result of the proposed method is shown in Figure 17, while the interpolated contour plots of three points are shown in Figure 17b–d, respectively. It can be seen that each target is well-focused with the proposed approach; corresponding performance indicators for measuring imaging quality, including resolution (res.), peak-side-lobe ratio (PSLR) and integrated-side-lobe ratio (ISLR), are computed and listed in Table 2. PSLR represents the ratio of the main lobe peak intensity to the maximum side lobe peak intensity, and ISLR represents the ratio of side lobe energy to main lobe energy. All simulation results in Figure 16 and Figure 17 and Table 2 validate the proposed azimuth pre-processing method to handle the raw data of squint sliding-spotlight SAR with BV-PRF for azimuth data uniform resampling capacity.

**Figure 18:**
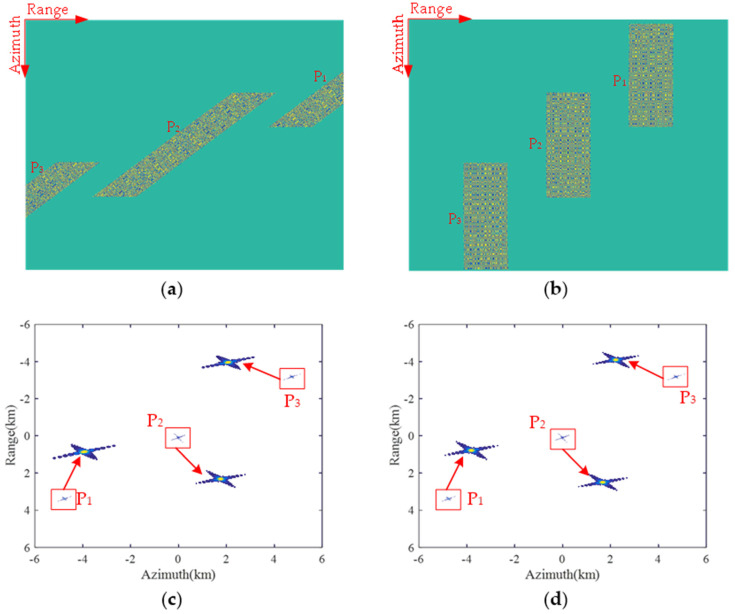
Simulation results of the fixed PRF and CV-PRF schemes. (**a**) The real part of echo data with the fixed PRF scheme; (**b**) the real part of echo data with the CV-PRF scheme; (**c**) imaging results with the fixed PRF scheme; (**d**) imaging results with the CV-PRF scheme.

The simulation results of fixed PRF and CV-PRF schemes are shown in Figure 18. As shown in Figure 18a, in the fixed PRF scheme, the echo data of P_1_ and P_3_ targets located in the edge of the scene cannot be completely obtained. Therefore, the resolution of P_1_ and P_2_ targets in imaging results decreases as shown in Figure 18c. The raw data of the whole scene can be successfully obtained by the CV-PRF scheme, as shown in Figure 18b, and the three targets are also well-focused in Figure 18d. However, the computational complexity of the CV-PRF scheme is approximately dozens of times greater than that of the proposed method. Therefore, the proposed approach is more effective.

## 6. Discussion

For the large range swath and azimuth scanning angle, targets located in the edge of the swath cannot be fully obtained by the fixed PRF due to the large RCM. Therefore, for the small scene and scanning angle, the fixed PRF scheme is more appropriate.

The CV-PRF can disperse the position of the blind areas due to transmitted pulses along the azimuth, so the skewed SAR raw data are completely rectified. Therefore, the CV-PRF scheme is suitable for the large imaging swath and azimuth scanning angle. However, the duration of the instantaneous echo receiving window is long in many imaging modes. The BV-PRF scheme can not only solve large RCM, but also greatly reduce the non-uniformity of azimuth sampling. Therefore, for the large imaging scene and azimuth scanning angle, the echo data of the whole imaging scene can be successfully obtained by BV-PRF scheme, and the subsequent azimuth data reconstruction also becomes efficient.

## 7. Conclusions

An azimuth full-aperture processing method for processing squint SAR raw data formed by BV-PRF scheme is proposed, which makes the whole raw data set have a sufficient and uniform azimuth sampling frequency. In a large imaging scene and azimuth scanning angle, the raw data of the whole swath with BV-PRF scheme can be completely obtained using a limited number of PRFs. Therefore, the BV-PRF scheme can be preferably used in spaceborne squint sliding-spotlight mode. However, when the number of samples in the designed BV-PRF scheme is too small and too large at the same time, there can be redundant operations in the proposed azimuth full-aperture processing method. In future research, an equal and sufficiently small sample number in the BV-PRF scheme should be designed. The de-ramping operation can be omitted in the proposed approach, which further reduces the calculation of the system. Furthermore, azimuth sub-aperture processing is also a strategy for processing the echo data generated by the BV-PRF scheme.

## Figures and Tables

**Table 1:** Simulation parameters.

Parameter	Value
Relative platform velocity	7212 m/s
Slant range of the scene center	750 km
Carrier frequency	5.6 GHz
Azimuth antenna length	6.3 m
Number of PRFs	3
Azimuth beam rotation rate	2.78°/s
Middle squint angle	25°
System PRF	2462/2511/2562 Hz
Pulse bandwidth	150 MHz
Range sampling frequency	200 MHz
Pulse duration	8 μs

**Table 2:** Performance indicators of three point-targets of the proposed method.

Target	Azimuth			Range		
	Res. (m)	PSLR (dB)	ISLR (dB)	Res. (m)	PSLR (dB)	ISLR (dB)
P_1_	2.87	−13.27	−10.19	0.94	−13.25	−10.10
P_2_	2.86	−13.25	−10.18	0.94	−13.25	−10.11
P_3_	2.76	−13.26	−10.18	0.94	−13.28	−10.12

## Data Availability

No applicable.
